# Knowledge of surgical informed consent and associated factors among patients undergone obstetric and gynecologic surgery at Jimma Medical Center, Jimma, Ethiopia, 2020: an institutional based cross-sectional study

**DOI:** 10.1186/s13741-023-00295-2

**Published:** 2023-03-16

**Authors:** Belete Fenta Kebede, Tsegaw Biyazin Tesfa, Aynalem Yetwale Hiwot, Yalemtsehay Dagnaw Genie

**Affiliations:** 1 Faculty of Health Sciences, Institute of Health, Jimma University, Jimma, Ethiopia; 2 Department of Nursing, College of Health Sciences, Mizan-Tepi University, MizanTeferi, Ethiopia

**Keywords:** Knowledge, Informed consent, Surgical patients, Jimma, Ethiopia

## Abstract

**Introduction:**

Informed consent is the process whereby a patient makes a voluntary decision about their medical and surgical care with knowledge of the benefits and potential risks. Poor informed consent processes may increase potential for medical errors and malpractice. Little is known of the knowledge of surgical informed consent with regard to their surgical treatment in Ethiopia. Therefore, this study aimed to assess the knowledge of surgical informed consent and associated factors among patients who underwent obstetric and gynecologic surgery at Jimma Medical Center, Jimma, Ethiopia.

**Methods and materials:**

An institution-based cross-sectional study was conducted from April 1 to May 30, 2020, among 404 women undergo obstetric and gynecologic surgery at Jimma Medical Center. Data were collected through a face-to-face interview using a structured questionnaire. The collected data were coded, entered into Epi data version 3.1, and analyzed using SPSS version 25. Bivariate and multivariate regression analyses were performed to determine the association between an outcome variable and an independent variable. Tables, pie-charts, and texts were used to report the result.

**Results:**

Of 404 patients sampled, only 372 women were agreed and participated in the study and gave response rate of 92.1%. The respondent satisfaction level (AOR 1.823 (95%CI 1.061–3.134)) and patient to provider relationship (AOR 0.472 (CI 1.217–3.697)) were associated with knowledge of surgical informed consent.

**Conclusion:**

The overall level of knowledge regarding informed consent for surgerywas significantly lower than that of other national and international figures. Patient satisfaction and patientto provider relationships were associated with knowledge of surgical informed consent. Adequate information should provide before surgery to improve patients’ knowledge regarding surgical informed consent and to improve the consent process to make it better suited to fit the needs of all patients.

**Supplementary Information:**

The online version contains supplementary material available at 10.1186/s13741-023-00295-2.

## Introduction

The concept of informed consent is an important aspect of biomedical ethics, which refers to the autonomy of patients in healthcare service providers and patient relationships (Bhute 2017). It is an ethical obligation of health care professionals to uphold patients’ autonomy and let them decide on the proposed medical, surgical, or other health care and research interventions (Lennox and Wright 2019). Consent is generally obtained in written form, but in some cases, it may be verbal especially for non-invasive and relatively non-risky interventions (Bhute 2017, Erkan et al. 2017).

Several factors may affect surgical informed consent, including patients competence, provision of limited information, unsuccessful communication between patients and professionals, hospital environment, privacy issues, and inadequate time (Bhute 2017, Lennox and Wright 2019, Teshome et al. 2018). The surgical informed consent process isvital to patients,however, patients vary in their views of its purpose with the dominant view enabling patients’ self-decision-making (Hammami et al. 2014).

Women need consistent and adequate information for consent and should undergo it with proper knowledge, awareness, and confidence (Tejaswi et al. 2020). Lack of knowledge on surgical informed consent increases the likelihood of a patients safety incident, patient anxiety, and postoperative dissatisfaction (Mbonera and Chironda 2018). Providing educational programs to patients is mandatory to fill knowledge gaps and improve the quality of the informed consent process (Galal 2016). Different studies in several countries showed diverse distribution level of knowledge on the subject of consent (Mbonera and Chironda 2018; Profile 2017; Viegas and Caregnato 2016). A study conducted among surgical patients at the hospital of Istanbul university of women declared that 38.1% of women they have not sufficient knowledge (Profile 2017), and a study in Brazil revealed only 44.7% of participants had full understanding of surgical informed consent (Viegas and Caregnato 2016). Similarly, a study conducted at Rwanda military referral hospital showed that 83%of women had low knowledge of surgical informed consent (Mbonera and Chironda 2018). Patients’ level of understanding on surgical informed consent was significantly associated with educational level (Abebe 2020; Convie et al. 2020) and other related factors of residency (Convie et al. 2020).

Currently, the importance of obtaining surgical informed consent before surgery is well established in all hospitals; however, the practice and knowledge of the surgical informed consent are not uniform among patients undergone obstetric and gynecologic surgery (Gebremedhn & Lemma 2014). Again, there is no enough studies on the patient’s level of knowledge towards the surgical informed consent and associated factors in Ethiopia including this study area. Therefore, this study aimed to assess knowledge of surgical informed consent and associated factors among patients undergone obstetrics and gynecologic surgery in Jimma Medical Center.

Assessment of knowledge of surgical informed consent and associated factors is important to set recommendations to improve the patient’s level of knowledge and to improve clinical practice in obstetrics and gynecologic procedures. This study will also help in research to conduct further studies and guide the development of evidence-based health care/practice.This has implications for the knowledge base generated and the relevance of policy initiatives to address knowledge gaps in maternal surgical informed consent.

## Methods and materials

### Study area and period

This study was conducted in the Jimma Medical Center, Jimma town, Ethiopia, from April 1 to May 30, 2020. Jimma town is situated approximately 354 km from Addis Ababa, the capital city of Ethiopia. The referral hospital is Jimma Medical Center (JMC) is one of the oldest public hospitals in the country. It was established in 1937 by Italian invaders. The hospital provides services to a population of 15–20 million in Oromia, Gambella, South West, and Benishangul regions. Some patients travel from South Sudan for treatment. The hospital has 1800 staff members, including 550 nurses. It has 800 beds and a 32-bed intensive care unit.

It provides many services under many departments, such as the pediatric, medical, surgical, maternity ward, obstetrics, gynecology, maternal and child health (MCH) ward, operating room (OR), emergency outpatient department (OPD), delivery room, psychiatric clinic, dental and eye clinics, laboratory service, ultrasound, and other imaging studies. Approximately 1461 and 900 patients underwent obstetric and gynecologic-related surgery within the past 6 months, respectively (the previous 6-month hospital report) from two oprating rooms (one obstetrics and one gyneclogy).

### Study design

An institutional-based cross-sectional study was conducted among patients admitted to the gynecologyand obstetrics ward of Jimma Medical Center, Jimma, Ethiopia.

### Study population

Women who underwent obstetrics and gynecologic surgeries in Jimma Medical Center were the source population. All selected women who undergone obstetrics and gynecologic surgeries were the study participants of the study.

### Eligibility and exclusion criteria

All women aged 18 years and above who undergo obstetrics and gynecologic surgery at Jimma Medical Center during the study period. Able to provide consent for the study. Women who were unable to consent to enter the study, such as not fully conscious at the time of data collection were excluded.

### Sample size determination

The sample size was determined using single population proportion formula by considering 50% proportion (P), because of no study conducted previously in Ethiopia; with 95% confidence interval (1.96), *α* = 0.05 and 5% marginal of error.

$$=\underset{}{\left(1.96\right)^2\times0.5\left(1-0.5\right)}/\left(0.05\right)^2=0.954/0.0025=384$$

After adding a non-response rate of 5% ( 19.2 ~ 20), the final sample size was 404.

### Sampling techniques and procedures

A systematic sampling technique was conducted to select study participants from the 798 total 2-month surgical cases after determining the interval (*K*th). The K-interval was determined by dividing the total 2-month surgical case (798) by the final sample size (404) which was approximately two. The first study participant was selected by the lottery method using their registration serial number and the rest were selected every at every interval from the registration book until the final sample size was reached.

### Variables

#### Dependent variable (outcome variable)

- ✓ Knowledge towards surgical informed consent

#### Independent variables

- ➢ **Sociodemographiccharacteristics:** age, educational status, occupation, marital status, and residence
- ➢ **Obstetricsand gynecologic related characteristics:** parity, type of surgery, and history ofprevious surgery
- ➢ **Health care providers related factors:** patient to health care provider relationship
- ➢ **Health facility-related factors/service-related factors:** the language of the written consent form, profession who requested informed consent, the timing of consent, time taken to provide informed consent, and time taken to make decisions.

### Operational definitions

#### Informed consent

The practice of providing necessary information that allows informed consent to make autonomous authorization.

##### Knowledge of surgical informed consent

In the context of this study, knowledge about surgical informed consent was considered “good” if the women answered knowledge questions with the above mean score. Knowledge about surgical informed consent was considered “poor” if knowledge questions were below the mean score (Mbonera and Chironda 2018).

#### Patient to doctor relationship

The patient to doctor relationship can be seen as the perception of the patient concerning the caring shown by the doctor and the attitude and behavior of the doctor towards the patient. The patient to doctor relationship questionary (PDRQ) contains nine items with yes/no options.

#### Data collection tools and procedures

Data were collected postoperatively before hospital discharge using pretested structured questionnaire by a researcher trained to conduct the interview. The questionnaire was developed after reviewing the published on surgical consent. The questionnaire consisted of socio-demographic factors consisting of 8 items, patient-related factors, health facility-related characteristics, patient to healthcare provider relationship and the last part dealswith patients’ knowledge of surgical informed consent. The tool was designed in English and Oromifa. Two BSc. nurses and one MSc. nurse were recruited as data collectors and supervisors, respectively.

#### Data quality assurance

Different measures were taken to ensure the quality the data. The questionnaire was initially prepared in English, translated into the local language (Afaan Oromo), and then translated back to English. Before the actual data collection, a pretest was conducted on 5% of the total samples in another setting. After obtaining the results, appropriate corrections were made before using them for the main study. A day of training was provided to the data collectors. During the data collection period, the principal investigator checked the data for completeness and consistency of information.

#### Data processing, analysis, and presentation

The collected data were coded and entered into Epi data 3.1 and exported to SPSS version 25 for analysis. The Hosmer-Leme show test was done to confirm the model fitness, and the model was fitted. Bivariate and multivariable analyses were performed between knowledge of surgical informed consent and the independent variables. In bivariate logistic regression, the variables which had a *p* value of less than 0.25 was considered as candidate variables for multi-variable logistic analysis. In multivariable analysis, those variables with a *p* value of less than 0.05 were considered statistically significant with the outcome variable. The findings of the data are presented using text, tables, figures, and graphs.

## Results

This study used to measure the level of knowledge towards surgical informed concept among participants to their surgical procedure, from the total sample of four hundred four (404) women, at three hundred seventy-two (372) of them were agreed to involve in the study and gave response rate of 92.1%.

### Socio-demographic characteristics

Three hundred seventy-two patients were recruited to the study. The median age of the respondents was 28 with an interquartile range (24–30) years. The majority of the respondents (83.3%) were married. Two hundred ninety-one respondents were literate (Table 1).

**Table 1:** Socio-demographic characteristics at Jimma Medical Center, Jimma, Ethiopia, 2020

Charactrstics	Category	Frequency	Percent
	< 20	31	8.3
Age (years)	20–35	293	78.8
	≥ 35	48	12.9
	Median	28 IQR 24–30	
Religion	Muslim	182	48.9
	Orthodox	123	33.1
	Protestant	64	17.2
	Others	3	.8
Educational status	lliterate	81	21.8
	Elementary completed school	121	32.5
	Secondary completed school	113	30.4
	Diploma and above	57	15.3
Maternal residency	Urban	242	65.1
	Rural	130	34.9
Marital status	Single	62	16.6
	Married	310	83.3
Occupation	Housewife	168	45.2
	Private employee	33	8.9
	Government employee	68	18.3
	Merchant	29	7.8
	Farmer	56	15.1
	Student	18	4.8

### Obstetrics and gynecology-related characteristics

In this study, one hundred sixty (43%) participants had priemigravida. Of the 372 women, two hundred seventy seven (74.5%) underwent emergency surgery andninty five (25.5%) of the women underwent elective surgery. A significant percentage, three hundred thirty (88.7%) and two hundred sixty-nine (72.3%) of participants had no previous medical and surgical history respectively. Two hundred twelve (57%) of the respondents were dissatisfied with the explanation given to them before the surgery and informed consent. The overall practice of informed consent was poor which accounting for two hundred thirty-seven (63.7%) of the study (Table2).

**Table 2:** Obstetrics and gynecology-related characteristics of knowledge towards informed consent at Jimma Medical Center, Jimma, Ethiopia 2020

Variable	Category	Frequency	Percentage
Parity	Nulli Para	14	3.76
	multi para	310	83.33
	Grand multipara	48	12.903
Type of surgery	Elective	95	25.5
	Emergency	277	74.5
Previous medical history	Yes	42	11.3
	No	330	88.7
Previous surgical history	Yes	103	27.7
	No	269	72.3
Number of operation done	1	60	58.3
	≥ 2	43	41.7
Satisfaction	Dissatisfied	212	57.0
	Satisfied	160	43.0

### Respondents’ knowledge on informed consent

The respondents who respond more than a mean score (4.8) for knowledge questions were considered as knowledgeable, and those who respond below the mean score (4.8.) were considered as having poor knowledge towards surgical informed consent. The mean score was 4.8 (Table 3).

**Table 3:** Respondents’ knowledge towards informed consent at Jimma Medical Center, Jimma, Ethiopia 2020

Questioners	Frequency and percentage	
	**Yes**	**No**
Signing the consent form is a legal requirement	268 (72%)	104 (28%)
Signing the consent form does remove your right to Compensation	216 (58.1%)	156 (41.9%)
Have the right to change your mind after signing consent?	74 9 (19.9%)	298 (80.1%)
If you are not able to sign the consent form, the operation couldn’t do?	285 (76.6%)	87 (23.4%)
If you refuse to sign the consent form, the operation couldn’t do even you die?	293 (78.8%)	79 (21.2%)
If you can't sign the consent form, your next of kin can sign on your behalf?	319 (85%)	53 (14.2%)
After consent, the doctor can do anything different from what was on the form as s/he wants?	289 (77.7%)	83 (22.3%)
A Doctor cannot do anything different from what was on the form unless it is lifesaving?	342 (91.9%)	30 (8.1%)

Knowledge items 2nd and 7th should be reversed for interpretation

**Fig. 1:**
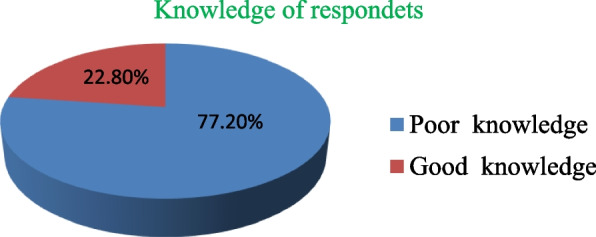
Displays the patient’s level of knowledge towards informed consent for surgical procedures at Jimma Medical Center, Jimma, Ethiopia 2020

Figure 1:- Displays the patient’s level of knowledge towards informed consent for surgical procedures at Jimma Medical Center, Jimma, Ethiopia 2020. In this study, the respondents had overall knowledge of surgical informed consent 287 (77.2% (95% CI 72.8–81.2)) was poor and only 22.8% of respondants had good knowledge.

### Health facility-related factors/service-related factors

In this study, 92.2% of the respondents had insufficient time to decide the procedure, and the majority of respondents (220), the mode of decision-making was self-method of decision. The majority (81.2%) participants reported having received surgical informed consent (SIC) counseling from resident physicians, the remaining got from obstetrician-gynecologists 44 (11.8%), and nurse-midwives 26 (7%) (Table 4).

**Table 4:** Health facility-related factors of patient’s knowledge at Jimma Medical Center, Jimma, Ethiopia 2020

Variable	Category	Frequency	Percent
Is consent form written with mother tongue	Yes	234	62.9
	No	124	33.3
Consent requested by	Ob-gyn specialist	44	11.8
	General practitioner/resident	302	81.2
	Midwife/nurse	26	7.0
Timing of consent	The day before the date of surgery	65	17.5
	On the day of surgery	87	23.4
	Immediately before surgery	208	55.9
	On the operation table	12	3.2
Time taken to provide informed consent	< 5 min	231	62.1
	5 -10 min	76	20.4
	> 10 min	65	17.5
Timing taken to decision making	Early	343	92.2
	Late	29	7.8

### Factors associated with knowledge of surgical informed consent

The association between the independent and dependent variables (outcome) was analyzed using a binary logistic regression model. In the bivariate analysis factors such as age, educational status, marital status, residence, schedule of surgery, satisfaction level, and patient-healthcare provider relationship were significant with a *p* < 0.25. Candidate variables inthe bivariate analysis were transported using multivariate logistic regression. Finally, level of satisfaction and patient healthcare provider relationship were found to be associated with knowledge regarding surgical informed consent in multivariate analysis with *p* < 0.05 (Table 5).

**Table 5:** Results of the bi-variable and multivariable logistic regression analysis of associated factors of patient knowledge towards surgical informed consent for surgical procedures at Jimma Medical Center, Jimma, Ethiopia/2020

Variable	Category	Knowledge		COR	*p* value	AOR	*p* value
		Good	Poor				
Age	< 35	69	255	1		1	.903
	> 35	16	32	1.004 (.488–2.066)		.955 (.454–2.006)	
Educational status	Illiterate	15	66	1		1	
	Literate	70	221	718 (.385–1.336)		.916 (.535–1.567)	.748
Marital status	Single	19	43	1		1	
	Married	66	244	.612 (.334–1.121)		1.821 (.974–3.407)	.060
Residence	rural	61	181	1		1	
	Urban	24	106	.672 (.396–1.141)		.690 (.401–1.186)	.179
Type of surgery	Elective	26	69	1		1	
	Emergency	59	218	.718 (.421–1.226)		.729 (.420–1.267)	.263
Satisfaction level	Dissatisfied	43	169	1		1	
	Satisfied	42	118	1.399 (.861–2.274)		1.823 (1.061–3.134)	.030*
Patient healthcare provider *r*/*n*	Poor	51	142	1		1	
	Good	34	145	.653 (.399–1.068)		2.121 (1.217–3.697)	.008*

*NB* 1 considered as reference categories, *COR* crude odds ratio, *AOR* adjusted ods ratio

In this study, satisfied patients were 1.8 times more knowledgeable regarding surgical informed consent than dissatisfied patients (AOR 1.823 (95%CI 1.061–3.134). Those patients who had poor relationships with health care providers were less likely to be knowledgeable about surgical informed consent (AOR 0.472 (95% CI 1.217–3.697).

## Discussion

This cross-sectional study was conducted to determine the level of knowledge regarding surgical informed consent and the factors associated with obstetrics and gynecologic surgery. The study showed that there was a significantly low (poor) level of knowledge 77.2% towards informed surgical consent. This was similar to a cross-sectional study conducted at Cairo University Hospital, Egypt, where 72.7% of participants had a poor level of knowledge regardingsurgical informed consent (Galal 2016).

However, the results of our study were lower than those studies conducted atRwanda (Mbonera and Chironda 2018), where 83% had low knowledge and St. Paul’s Hospital Millennium Medical College, Ethiopia (Abebe 2020) where approximately 89.5% participants had poor level of knowledge regarding surgical informed consent. The Rwandan study (Mbonera and Chironda 2018) had a different inclusion criteria, and participants were from all surgical departments (general surgery,orthopedic, obstetrics and gynecology, urology, ENT, maxilla facial, and plastic surgery. The study conducted at St. Paul’s Hospital Millennium Medical College, Ethiopia (Abebe 2020), had a different in sociodemographic population, and it included both men and women.

Our study found higher level of knowledge about surgical informed consent than the cross-sectional studies conducted at a dental hospital in rural Haryana in India 17% (Singh 2012), at Istanbul University, Turkiye 38.1% (Profile 2017), and at Aminu Kano, Nigeria, 2.5% (Sulaiman et al. 2015) participants had poor level of an overall knowledge regarding surgical informedconsent. Again this may represent different surgical populations.

In addition, this study revealed that higher professional and patient relationship experience was associated with knowledge of informed surgical consent, and those patients who hadpoor relationships with health care providers were less likely to be knowledgeable than their counterparts. This was supported by another study conducted in Lowa City, Iowa (Xu and Prince 2019), and a systematic review on patient and doctor relationships (Chandra 2018). This is because shared decision-making improves the caregiver/patientrelationship and integrates the patient as an actor in his/her care. It also modifies the patient/physician relationship in the surgical field (Code and Health 2019).

Patient satisfaction in the level of the surgical informed consent process was associated with knowledge regarding surgical informed consent, satisfied patients. This is similar to studies conducted at the German university hospital (Weckbach et al. 2016) and the American College of Rheumatology (Johnson et al. 2011). This may be due to patients having higher satisfaction if their surgical informed consent was readand understood by thepatients. Preoperatively patients who are satisfied with the consent process may have better recall of the risks and benefits of surgery. Our study demonstrated that knowledge of surgical informed consent increases satisfaction and is associated with a better patient-health care provider relationship.

## Conclusion and recommenedation

Knowledge of patients towards surgical informed consent for their obstetric and gynecologic surgical procedures was significantly low in this study. Factors such as patient health care provider relationship and satisfaction level were associated with knowledge towards surgical informed consent. The hospital needs to design knowledge improvement measures towards surgical informed consent for patients. Adequate information should be givento the patient orally and in writing during hospitalization to improve knowledge towards surgical informed consent. Surgeons should make out adequate time to explain surgical procedures and possible complications to patients before any surgical procedure to improve knowledge ofsurgicalinformed consent.

### Limitation of the study

The possible limitations of the study are as follows: both emergency and elective obstetrics and gynecologic patients were included. Emergency surgery respondents probably would be less informed than elective patients considering the stress involved and time constraints in emergency procedures. Clients often rush into surgery before discussing the issue with family and friends. Previous studies have found that clients undergoing elective surgeries, which gave them enough time to make informed decisions achieve a better understanding of proposed treatments compared with clients undergoing emergency surgeries, where there is little or no time for a lengthy discussion.

## Supplementary Information

**Additional file 1. Annex: Questionary.**

## Data Availability

The datasets are obtained from the corresponding author upon reasonable request.

## Abbreviations

CI: Confidence interval
COR: Crude odds ratio
JMC: Jimma Medical Center
OR: Odds ratio
PDRQ: Patient-Doctor Relationship Questioner
SD: Standard deviation
SIC: Surgical informed consent
