# Supplementary material for: Knowledge of surgical informed consent and associated factors among patients undergone obstetric and gynecologic surgery at Jimma Medical Center, Jimma, Ethiopia, 2020: an institutional based cross-sectional study
Source: Perioper Med (Lond). 2023 Mar 16;12:6. doi: 10.1186/s13741-023-00295-2 (PMC10018955; doi:10.1186/s13741-023-00295-2)
Supplement: Supplementary file 1 — Additional file 1. Annex: Questionary. [file 13741_2023_295_MOESM1_ESM.docx]

Annex : **Questionary**

| ***Part I: Socio-demographic characteristics*** | |
| --- | --- |
| 1. Age in completed years | ____________ |
| 1. What is your educational level? | 1. No formal education 2. primary (grades 1-8) 3. Secondary and preparatory (Grades 9-12) 4. college and above |
| 1. What is your marital status? | 1. Single 2. Married 3. Divorced 4. Widowed |
| 1. What is your religion? | 1. Christian Orthodox 2. Protestant 3. Muslim 4. Catholic 5. Others specify_____ |
| 1. Where is your region? | 1. Oromo 2. Amhara 3. Tigre 4. Gurage 5. Other specify__________ |
| 1. What is your occupation? | 1. Housewife 2. Private employee 3. Government employee 4. Merchant 5. Farmer 6. Others specify___________ |
| 1. What is your residence? | 1. Urban 2. Rural |
| 1. What is your Regular monthly income? | ______ EB |

| ***Part II: Obstetrics and gynecology related characteristics*** | |
| --- | --- |
| 1. How many children do you have? | 1. One 2. Two to four 3. Greater than five 4. Null |
| 1. What is the type of surgery performed?   (in terms of time) | 1. Elective 2. Emergency |
| 1. What is the type of present surgery?   (document review) | 1. Benign tumor 2. Gynecological Ca 3. Fistula 4. Obstetrics C/S 5. Other specify_______ |
| 1. History of pervious medical diseases | 1. DM 2. HNT 3. Cardiac 4. Other specify_________ 5. None |
| 1. Do you have previous operation? | 1. Yes 2. No |
| 1. If yes how many number of previous operations do you have | 1. 1 2. ≥ 2 |

| ***Part III: Health facility-related factors*** | | | | |
| --- | --- | --- | --- | --- |
| 1. Do you referred from other health facility? | | 1. Yes 2. No | | |
| 1. Is the informed consent form available? | | 1. Yes 2. No | | |
| 1. If Q2 yes-How informed consent form written? | | 1. In my mother tongue 2. Not in mother lounge | | |
| 1. What is the status of profession of the person who gave surgical informed consent? | | 1. Obstetrician-gynecologist 2. Resident physician 3. Nurse-midwife 4. Did not know | | |
| 1. When was the timing of counseling for informed consent? | | 1. The day before date of surgery 2. On the day of surgery 3. Immediately before surgery 4. On the operation table | | |
| 1. Time elapse to give informed consent? | | 1. Less than 1 hour 2. Greater than 1 hour | | |
| 1. Time spent on the consent process | | 1. Less than 5 minute 2. 5 to 10 minute 3. Greater than 10 minute | | |
| 1. Who sign Consent form? | | 1. Self 2. Parent 3. Spouse 4. Physician 5. Other____________ |  |  |
| 1. Satisfaction with the consent and other services | | 1.Dissatisfied  2.Satisfied |  |  |
| 1. Patient healthcare provider r/n | | 1.Good  2.poor |  |  |

| ***Part iV: Patient’s knowledge to wards surgical informed consent*** | | | |
| --- | --- | --- | --- |
| No | Items | yes | No |
|  | Signing the consent form is a legal requirement? |  |  |
|  | Signing the consent form does remove your right to Compensation? |  |  |
|  | Have the right to change your mind after signing consent? |  |  |
|  | If you are not able to sign the consent form, the operation couldn’t take place, even if this means you could die? |  |  |
|  | If you refuse to sign the consent form you could die? |  |  |
|  | If you can't sign the consent form, your next of kin can sign on your behalf? |  |  |
|  | After consent the doctor can do anything different from what was on the form as s/he wants? |  |  |
|  | Doctor cannot do anything different from what was on the form unless it is lifesaving? |  |  |
